# The expansion of gambling across the Americas poses risks to mental health and wellbeing

**DOI:** 10.1016/j.lana.2024.100855

**Published:** 2024-07-29

**Authors:** Daria Ukhova, Virve Marionneau, Rachel Volberg, Heather Wardle

**Affiliations:** a School of Social and Political Sciences, University of Glasgow, Glasgow, United Kingdom; b Centre for Research on Addiction, Control and Governance (CEACG), University of Helsinki, Helsinki, Finland; c School of Public Health and Health Sciences, University of Massachusetts Amherst, Amherst, MA, USA

**Keywords:** Gambling, Gambling harms, Mental health, Mental health policy, PAHO

## Abstract

The Americas are facing a significant burden of mental health conditions. The Pan American Health Organisation’s regional Strategy for Improving Mental Health and Suicide Prevention is an important milestone in tackling this challenge. However, absence of any focus on gambling as a potential risk to the health and wellbeing represents a serious omission in the Strategy. In this viewpoint, we review existing scholarship demonstrating unequivocal links between gambling and a variety of mental health conditions and related harms. This is followed by an empirically-grounded discussion of how commercial gambling has recently rapidly expanded across the region and how the risks of this expansion have not been sufficiently considered at the policy level. We then present emerging regional evidence of the negative mental health impacts of gambling expansion. The review concludes by proposing possible policy actions to improve control over the gambling industry and reduce ensuing harms on mental health and wellbeing in the region, with a focus on PAHO’s remit.

## Introduction

The Americas are facing a significant burden of mental health conditions.^1^^,^^2^ The high burden of these conditions, combined with low treatment coverage makes mental health a serious public health issue for the region. The COVID-19 pandemic has worsened many pre-existing mental health conditions, including depressive and anxiety disorders. The Americas are now the only World Health Organisation (WHO) region where suicide rates are increasing.^2^

Recent strategies introduced by the Pan-American Health Organisation (PAHO) have tried to address the lack of prioritisation and underfinancing of treatment and prevention of mental health issues in the region.^1^^,^^2^ The approved regional Strategy for Improving Mental Health and Suicide Prevention^2^ and the New Agenda for Mental Health in the Americas^1^ identify a range of social determinants related to mental health outcomes and substantial barriers to treatment. These include significant inequities related to systemic racism, poverty, gender inequality, and stigma. Alcohol use is identified as a driver of suicidality,^1^ and alcohol use disorder is seen as a key mental health issue in the region.^2^ However, neither document identifies gambling as a potential threat to achieving the ambitions of building “*mental health systems and services that are equitable, resilient, and sustainable in the face of increasing and emerging threats to mental health*”.^1^

Gambling is consistently omitted from policies aiming to improve health and wellbeing, which risks undermining the ambitions of these policies. This risk is compounded by the rapid growth in commercial gambling, with both North and South America vastly expanding commercial gambling provision by legalising online and offline sports betting and expanding casino gambling. Whilst gambling is associated with a wide range of financial, cultural and crime-related harms, in this paper we focus specifically on mental health in response to the PAHO's omission of gambling from their mental health strategies. We outline five reasons why the PAHO and other governmental and intergovernmental agencies should view gambling expansion as an emerging threat to mental health and wellbeing and why it is essential to mainstream gambling harm prevention across all policies aiming to improve mental health within the Americas.

### Gambling disorder is a health condition and should be recognised as such

Gambling is recognised by the WHO as a health-harming activity. The ICD-11 recognises *Gambling Disorder* (GD) as a health disorder, *"characterised by a pattern of persistent or recurrent gambling behaviour"*.^3^ This is manifested by impaired control over gambling, gambling taking precedence over other life interests, and the continuation or escalation of gambling despite negative consequences. Diagnostic criteria for GD are available also in the DSM-5.^4^

The ICD-11 also identifies *hazardous gambling* as “*a pattern of gambling that appreciably increases the risk of harmful physical or mental health consequences to the individual or to others around the individual that may require intervention or monitoring but is not considered a disorder*”.^3^ This definition recognises that individuals may experience harmful consequences from gambling without meeting the diagnostic requirements for GD, which nevertheless may result in mental health and other harms to themselves and concerned significant others (CSOs).

Global synthesis of estimates suggests that around 1.41% of the adult population experience GD or problematic gambling (PG).^5^ In North America, 1.54% women and 2.69% of men in the general population are estimated to experience GD/PG.^5^ Regional-level estimate is not available for Latin America, but recent country-level prevalence rates for GD have ranged between 1.1% and 38.2%, with the highest rates measured in a student population in Brazil.^6^ Data on hazardous gambling has not been uniformly collated, though estimates of hazardous gambling are likely to be higher than those for GD, given its wider and sub-clinical definition. There is increasing evidence that the prevention paradox applies to gambling whereby a large proportion of harm from gambling at the population level occurs to people who do not meet clinical criteria for GD.^7^

There are significant gender inequities in GD. According to ICD-11, lifetime prevalence of GD is higher among men than among women, with an estimated ratio of 2:1.^3^ Recent evidence suggests that the gender gap in gambling participation and risks of harm might be narrowing, particularly, for younger age groups,^8^^,^^9^ thus contributing to worsening mental health outcomes among young women.

### Gambling is strongly associated with other mental health conditions, harms, and suicidality

The Americas have the highest prevalence of anxiety disorders and the second-highest rate of depressive disorders of all WHO regions.^2^ Alcohol consumption has a significant impact across the Americas, with 8.2% of adults estimated to be suffering from an alcohol use disorder.^2^ GD/PG co-occur with many other mental health conditions and other harmful consumptions. The relationships between GD and various other psychiatric and substance use conditions are well-established and include: alcohol use disorder, generalised anxiety disorder, major depressive disorder, and obsessive-compulsive disorder.^10^

The temporal sequencing between gambling and other co-occurring issues is subject to continuing debate.^11^ Gambling may both cause negative mental health outcomes and be a consequence of them, serving as a coping mechanism to escape physical or emotional problems. A longitudinal study of young adults in Canada indicated that at-risk gambling or PG represents a significant risk factor for the onset of mental health and substance use conditions (such as depression, alcohol use, and illegal drug use) rather than the reverse.^12^

Self-harm and suicide are two of the most severe gambling-related harms. GD is associated with increased risks of self-harm, suicidality, and suicide.^13^, ^14^, ^15^ Population-based studies show that suicidal thoughts and suicide attempts are prevalent among those experiencing GD.^16^^,^^17^ As with other co-occurring mental health issues, the temporal sequencing between gambling and suicidality is complex: for some, it may be explained by common etiologic factors while for others, gambling may be a dominant factor in suicidality.^15^^,^^18^ Qualitative evidence suggests that gambling may precede many comorbidities such as depression, with research linking suicidal behaviour or suicidal ideations directly to gambling.^14^

Hazardous gambling is linked to a range of harms beyond mental health issues which may, nonetheless, interact to promote poorer mental and physical wellbeing for individuals and their families. These include financial detriment and indebtedness, adverse effects on work and education, and severe harm to family life.^19^ Mental health impacts and other harms also accrue to others.^20^ Increased relationship strain, financial debt, and domestic violence associated with gambling all negatively affect CSOs’ mental health and wellbeing. An individual experiencing GD/PG is estimated to affect approximately six CSOs,^21^ thus the negative impacts of gambling span far beyond the individual engaging in gambling activity.

### The rapid expansion of commercial gambling is likely to increase attendant risk of mental health harms

Expansion of gambling provision forms part of the wider aetiology of GD and hazardous gambling.^22^ Throughout the Americas, the availability of gambling has increased exponentially. Except for Cuba, where all forms of gambling are illegal, all other nations across the Americas permit commercial gambling in some form.

Our analysis of regulatory changes in the Americas region, based on the Vixio Gambling Compliance Database,^23^ indicates that between 2018 and 2023, 31% of PAHO member states (11/35) passed legislation expanding access to gambling (often online but also land-based provisions). A further 11% (4/35) are likely to do this soon.

The legalisation of sports betting is a notable trend across the Americas:

• USA: In 2018, the US Supreme Court ruled to legalise sports betting. By March 2024, it has become available in 38 US states and the District of Columbia.
• Brazil: A new law regulating fixed-odds land-based and online sports betting was passed in 2018.
• Ecuador: ‘Sports forecasting’ was ruled legal by a state attorney in 2020, which effectively made online sports betting legal.
• Canada: Canada legalised single-game sports betting at the federal level in 2021.

The increasing availability of gambling has generated increased consumption and increased revenues for the commercial gambling sector. Data from the USA shows that Gross Gaming Revenue (GGR) from sports betting (the total amount lost by consumers once winnings have been paid) increased from less than US$1 billion in 2018 to over US$7.5 billion in 2022.^23^ Growth projections for Latin America suggest that GGR for online sports betting alone will increase from under US$1 billion in 2021 to over US$4 billion by 2026.^23^

In addition to the expansion of sports betting, there is also expanding access to casino-type gambling in land-based and online environments across the region. Casino gambling products are identified as particularly harmful to consumers.^24^ The introduction of online casino gambling may spread to new jurisdictions. As experience of change within the USA shows, once online sports betting is adopted, the next debate that the industry and others focus on is the legalisation of online casino games and slots.

Gambling industry lobbying activities are at the core of this expansion. Across the USA, gambling industry lobbyists have been shown to play a crucial role in crafting sports-betting legislation, exaggerating potential economic benefits without paying any attention to potential harms.^25^ Globally, research evidence shows that increased availability of gambling, and particularly harmful and intensive forms of gambling, leads to increased levels of harms, at least in the short to medium term.^26^^,^^27^ The relationship between availability and harm is commensurate with the total consumption model, where there is some evidence demonstrating that greater gambling consumption among the population is associated with higher rates of population harm.^28^ For example, analysis from the USA has noted a positive relationship between casino density and suicide mortality.^29^

Finally, the region houses some of the largest global offshore gambling license providers, including Antigua and Barbuda, Costa Rica, Curacao, and Panama. Other countries, such as Trinidad and Tobago, which have recently expanded legal gambling provisions, have aspirations to develop offshore gambling licensing. Offshore gambling refers to gambling provision that emanates from other jurisdictions and is unregulated or illegal in the targeted markets. The use of offshore gambling sites is associated with greater levels of harmful gambling,^30^ and contributes to the burden of gambling in the Americas and beyond.

Thus, the expansion of the gambling industry and increased gambling consumption across the Americas are important factors for heightened levels of mental health harm.

### Motivations for gambling expansion focus primarily on revenue generation, not health protections, with weak regulatory policy and oversight

A primary driver for expanding commercial gambling is to generate economic revenue and growth. In the USA, 38 states and the District of Columbia (N = 39) have now implemented major changes to their commercial gambling provision (including, but not limited to sports betting) since 2018. Based on a review of their legislation, 23 (58.9%) of these states have explicitly done so to increase revenues to the state or to promote economic growth and tourism. Across the USA, the revenues generated by gambling are being used to fund a variety of public services, including health, welfare, education, infrastructure, and policing. In some states, a small proportion of funds are dedicated to so-called compulsive gambling services. Nineteen (48.7%) states cite the need to promote public trust and preserve integrity in gambling as primary motivation. Only four out of 39 (10.2%) states have mentioned the protection of public health as a motivation for legislative change.

Revenues from gambling are regressive, disproportionately generated from those most socially and economically disadvantaged.^31^^,^^32^ The increasing reliance on these revenues to boost economic growth or to fund public service provision is deeply problematic and risks undermining progress towards several UN Sustainable Development Goals, including exacerbating rather than reducing inequalities and undermining trust in institutions.

Globally, research has highlighted major legislative disparities between the recognition that gambling may be harmful and the legislative actions proposed to prevent those harms. Ukhova et al. demonstrated that the focus of preventive action tends to be the individual, whereby it is deemed that the individual should be in control of their behaviours and should be provided with tools to self-manage their gambling.^33^ This intervention paradigm is favoured by industry actors, displacing focus away from the actions of industry and how corporate practices should be managed.^34^ While the efficacy of many individual-focused preventive policies has been questioned, yielding either small or null results, many jurisdictions currently reforming their gambling policies rely on these weak solutions to gambling-related harms, offering few protections to the increasingly exposed populace.

### Gambling harms are growing across the Americas

There is emerging evidence that the expansion of legalised gambling across the Americas is associated with increased rates of hazardous gambling and GD/PG, with attendant impact on the health and wellbeing of individuals and communities in this region. Evidence from Illinois, USA which recently expanded all forms of gambling, shows that PG prevalence rates are now 3.8%, markedly higher than the global and regional averages.^5^ In Ontario, Canada, a significant expansion of the online sports betting markets in 2021 led to a surge in help-seeking for online gambling problems and harms.^35^ Similar increases in calls to helplines were observed in Illinois and a range of US states that legalized sports betting.^36^ Evidence from an online mutual support group in the USA showed an immediate and significant increase in help-seeking after the introduction of online sports betting.^37^

In some jurisdictions, recognition of harms and their potential growth have led governments to implement more stringent regulation of gambling provisions. These include a moratorium on new casino licenses in Mexico in 2020 and a ban on non-casino electronic gambling machines (EGMs) in Paraguay, which was implemented primarily to protect children and adolescents from *"possible harms to physical and mental health"* caused by EGMs.^38^

## Conclusion

The expansion of gambling across the Americas represents a potential risk to the health and wellbeing of populations within those areas. Without strong regulatory controls, gambling can exacerbate a range of poorer mental health outcomes, including suicidality, and the expansion of gambling is associated with increased gambling harms. We have seen the same development in other jurisdictions globally, with increased availability connected to worsening harms and increased burdens on mental health.^39^

Thus, strong consideration should be given by those charged with reducing mental health inequities to the role of gambling in exacerbating mental health burdens across the Americas. In the Americas and elsewhere, the ethics of relying on the growth of gambling to fund public institutions and stimulate growth should be examined. Gambling worsens existing inequities but also risks undermining the trust, transparency, and resiliency of these public services through reliance on gamblers’ losses to fund public institutions.

Whilst it is incumbent on governments to enact and enforce strong regulatory policies, many governments are not engaging at this level, enacting weak policy controls that focus on responsibility of individuals.^33^ There is thus a notable gap in leadership, a gap which agencies like the PAHO can fill. This starts with mainstreaming gambling within and across all relevant plans and strategies, embedding an action across all policies approach. To improve control over the gambling industry and reduce ensuing harms on mental health and wellbeing, governmental and intergovernmental agencies in the region, including PAHO, should consider the following:

• Relevant regional initiatives, such as The New Agenda for Mental Health in the Americas, and country-level mental health strategies should recognise gambling as both an outcome of and risk factor for mental health issues.
• PAHO could support raising awareness of the public health implications of gambling with their members and provide technical guidance and support on these issues.
• A regional-level surveillance and monitoring mechanism is needed to understand the scale and scope of gambling harms and commercial gambling expansion across the Americas, with a particular focus on youth.
• PAHO Member States expanding gambling provisions should recognise the attendant risks to physical and mental health associated with this expansion. They should enact and enforce regulatory regimes which prioritise the protection of the health and wellbeing of populations; and move away from currently predominant individually-focused policies and interventions favoured by gambling industry actors towards systemic population-based gambling harm prevention approaches. Multisectoral and multilateral efforts should also be directed towards restricting and regulating offshore gambling licensing, including enforcement of player protection measures.
• Health systems in PAHO Member States should incorporate provision of universal support and treatment for gambling harms free at the point of use.

A critical opportunity to improve mental health outcomes across the Americas would be to address gambling as part of the implementation of the new regional Strategy for Improving Mental Health and Suicide Prevention.

## Contributors

DU and HW jointly conceptualised the paper. DU, HW, and VM developed the methodology. DU, VM, HW, and RV conducted data collection, coding, formal analysis, and curated the data. DU and HW wrote the first draft of the publication. All co-authors reviewed and edited subsequent drafts. DU managed the project on a day-to-day basis, and HW provided overall supervision.

VM had access to Vixio database and produced summaries of legislative changes for all countries in the Americas. DU, HW, and RV verified that data from open-access online sources. VM, DU, HW, and RV then agreed on case selection. DU and HW retrieved primary legislations for selected cases from open access online sources. DU, HW, VM, and RV had access to Excel database with coded excerpts of primary legislative texts and accessed and verified the underlying data.

DU had final responsibility for the decision to submit for publication.

## Declaration of interests

DU has been funded as a member of staff at the University of Glasgow to work on this project by Wellcome Trust through a Humanities and Social Sciences Fellowship and by the University of Glasgow through the Lord Kelvin Adam Smith Readership Fellowship to HW. VM has been funded for gambling studies by the Finnish Ministry of Social Affairs and Health (section 52 of the Finnish Lotteries Act), the Academy of Finland (Project 349589 CODEG; Project 31834 POLEG), the Finnish Foundation for Alcohol Studies, French Observatory on Drugs and Addictions, and the Finnish Ministry of Justice. VM declares consulting fees from the Gambling harms evaluation group under the Finnish Ministry of Social Affairs and Health. VM has received support for travel from the Finnish Foundation for Alcohol Studies, University of Bergen, and the Council of Europe, Pompidou Group. VM has been paid for delivering a webinar by Bochum University, and for peer reviewing by Routledge. She regularly provides expert advice and consultations to third sector and public sector across in Finland. RV has been funded for gambling studies by the Massachusetts Gaming Commissions, the University of MA Donahue Institute, Connecticut Department of Mental Health & Addictions Services, and Evergreen Council on Problem Gambling. RV declares consulting fees from Karolinska Institute, NatCen, and Greo. RV declares travel costs paid by International Gaming Conference 2023 and Alberta Gambling Research Institute.

In the past 5 years, HW has been funded for gambling studies by the Economic and Social Research Council, National Institute for Health Research, Wellcome Trust, the Gambling Commission (including their regulatory settlement fund), Office of Health Disparities and Improvements/Public Health England; Greater London Authority; Greater Manchester Combined Authority; Blackburn with Darwen Local Authority and the Department of Digital Culture Media and Sport. In 2018/19, HW has received funding from GambleAware for a project on gambling and suicide. HW declares consulting fees from the Institute of Public Health, Ireland and the National Institute for Economic and Social Research. HW declares payment for delivery of seminars from McGill University and from John Hopkins University. HW has been paid as an expert witness by Lambeth and Middlesborough Borough Councils; HW declares travel costs paid by Gambling Regulators European Forum, the Turkish Green Crescent Society, Alberta Gambling Research Institute and the REITOX Academy (administered through the Austrian National Public Health Institute). She served as Deputy Chair of the Advisory Board for Safer Gambling between 2015 and 2020, remunerated by the Gambling Commission; is a Member of the WHO panel on gambling (ongoing) and provided unpaid advice on research to GamCare for their Safer Gambling standard (until 2021). She runs a research consultancy for public and third-sector bodies only. She has not, and does not, provide consultancy services to the gambling industry.
